# Self-regulation, stress appraisal, and esport action performance

**DOI:** 10.3389/fpsyg.2023.1265778

**Published:** 2023-10-10

**Authors:** Michael G. Trotter, Emmanuel A. C. Obine, Benjamin T. Sharpe

**Affiliations:** ^1^Department of Psychology, Umeå University, Umeå, Sweden; ^2^Institute of Psychology, Business and Human Sciences, University of Chichester, Chichester, United Kingdom

**Keywords:** challenge, threat, self-regulation, stress, Counter-Strike, video-games, e-sport

## Abstract

Electronic sport has seen substantial growth in market value and popularity in the last 10 years. With this growth has come the pursuit of elite esports performance, especially from a psychological perspective. This study aimed to investigate potential variations in self-regulation levels among athletes of different levels (national vs. student), compare the self-regulation profiles of CS:GO players in the current study to an international sample of e’athletes and to assess the predictive capacity of self-regulation on performance outcomes. A total of 53 esports athletes (student competitors, *n* = 27 and national-level CS:GO competitors, *n* = 26), participated in an experiment exploring self-regulation, DRES, and action performance. Furthermore, analysis comparing our collective findings against a larger global sample of e’athletes (*n* = 993) was conducted. Results demonstrated that CS:GO players who displayed higher levels of self-regulation tended to perceive stressful situations as challenges, consequently showcasing superior accuracy and time trial performance. In contrast, individuals with lower self-regulation tended to perceive such situations as threats, which correlated with less favorable performance outcomes. On a broader scale, the study observed that CS:GO competitors generally exhibited lower levels of self-regulation when compared to the larger global sample. Furthermore, self-regulation was identified as a mediating variable in the relationship between stress appraisal and performance, suggesting that improved self-regulation skills can lead to enhanced accuracy and quicker time trial performance. This may imply that competitors with greater self-regulatory abilities perceive themselves as having more personal resources, enabling them to effectively assess challenging situations and employ problem-focused coping strategies. Overall, this research underscores the significance of self-regulation in optimizing esports performance, while providing valuable insights for player development, action performance, and overall outcomes in the field.

## Introduction

Sporting success requires constant cognitive, behavioral and emotional regulation, over a long period of time both during practice and in competitive settings (Crocker et al., 2015). To excel in competitive contexts, athletes must possess the ability to self-regulate their behaviors through meticulous planning, control, and adaptation (Jonker et al., 2010a; Young et al., 2023). In the realm of esports, research has revealed that esports athletes (e’athletes; Bubna et al., 2023) who exhibit strong self-regulation skills are more likely to achieve success (Trotter et al., 2021). Behnke et al. (2020) observed that experienced e’athletes who perceived themselves to possess abundant personal resources relative to the demands of the competition tended to appraise stressful situations as challenges rather than threats. To date, no research has explored the possibility that self-regulatory skills may influence stress appraisal in esports. The study aims to enhance our understanding of the relationship between self-regulation and stress appraisal in e’athletes. It is anticipated that self-regulatory skills will manifest as prominent personal attributes, particularly among expert e’athletes, and that these skills will mediate the connection between stress appraisal and performance.

### Esport performance

Achieving high performance is perceived as being paramount in the realm of professional sports culture (Douglas and Carless, 2006). This holds true for competitive esports, which boasts year on year growth of industry revenue at 16.4% in 2022 with the industry estimated to be worth over 24.9 billion US dollars (Ahn et al., 2020; Newzoo, 2022). Furthermore, the popularity of esports shows no signs of diminishing. Notably, performance in esports can significantly impact a team’s or individual’s ability to secure sponsorships, which are crucial for financing various competition-related expenses such as travel and obtaining vital support health services like psychology or physiotherapy (Hong, 2022). Sponsorships are also a vital component of the business models of many esports stakeholders (Scholz, 2019). The inability to deliver satisfactory performance may negatively affect a player or team’s prospects of acquiring or retaining sponsorships, as sponsors may be hesitant to associate their brand with notions of failure and inferiority (Crompton, 2015). Given the substantial impact of performance in the esports industry, it is not surprising that a significant amount of research has been dedicated to investigating factors that distinguish high-performing teams from lower-performing ones. Research has explored various performance related factors variables, including stress coping, utilization of psychological skills, self-regulation, and physical activity (Poulus et al., 2020, 2022c; Trotter et al., 2020, 2021).

Specifically, esports psychology research has shown significant interest in stress and coping (Leis and Lautenbach, 2020; Leis et al., 2021) psychological skill use (Trotter et al., 2021; Bonilla et al., 2022). Studies have highlighted notable internal stressors (e.g., performance pressures) and external stressors (e.g., performing in front of an audience; see Poulus et al., 2022a,b for qualitative investigations), that appear to inhibit performance. The assessment of esports performance often relies on broad indicators such as in-game rank, which may lack transparency regarding the calculation methods and the specific factors influencing rank assignment (Poulus et al., 2020; Trotter et al., 2020, 2021). A recent Delphi study conducted by Sharpe et al. (2023a) engaged esports performance experts to explore alternative measures of esports performance. The study highlighted the inclusion of more action performance measures (any performance measure that may directly or indirectly influence the outcome of the domain goal), such as reaction time and accuracy, as they offer a more comprehensive and reliable assessment of individual esports performance to date. These metrics present a promising avenue for future research in the field, offering valuable alternatives to relying solely on game rank and provide an opportunity for researchers to note the intention of their research more explicitly. As such, the following line of research will be investigating the influence of stress appraisal and self-regulation on esport action performance (see Sharpe et al., 2023a for description). Effective self-regulation is one potential action performance measure, which has been associated with performance in numerous contexts (Toering et al., 2009; Jonker et al., 2010a; Trotter et al., 2021).

### Self-regulation

Self-regulation can be defined as the cyclical “capacity to plan, guide, and monitor one’s behavior” (Brown, 1998, p. 62). Within the field of psychology, self-regulation has garnered significant attention, resulting in the formulation of various models since the 1980s. These models diverge in terms of the number and sequence of their phases and subprocesses (see Panadero, 2017 for a comprehensive review). While each self-regulation model possesses its unique characteristics, each model can generally be categorized as having three main phases: preparatory phase (e.g., task definition, planning, goal setting), performance phase (e.g., monitoring, control, utilization of task-specific strategies), and appraisal phase (e.g., performance feedback, reflection, adaptation, regulation). During the preparatory phase, individuals establish goals and select appropriate strategies based on the available information. In the performance phase, individuals enact their chosen strategies and assess the efficacy of the associated behaviors. Finally, in the appraisal phase, individuals reflect on goal outcomes and prepare for future performances.

The Miller and Brown (1991) and Zimmerman (2000) models of self-regulation have been previously used to measure self-regulation in esports (Kleinman et al., 2021; Trotter et al., 2021, 2022). This study has chosen to use the Miller and Brown (1991) model, which includes seven phases of self-regulation (i.e., information input, self-evaluation, instigation to change, search, planning, implementation and plan evaluation) to allow for comparison of this studies results, with quantitative self-regulation results from a previously measured global population of e’athletes (Trotter et al., 2021). In the Miller and Brown (1991) model, the self-regulation cycle begins with information input, which involves the monitoring of the self and external situational cues to determine the capacity for coping with situational demands. Information input is followed by self-evaluation, which occurs to judge if a disparity exists between a current state and a goal state. If a discrepancy exists (e.g., being in the process of losing a current esports match), then an instigation to change occurs to consciously adjust behaviors toward the goal. The individual then searches for possible options to reduce the discrepancy (e.g., changing the current game strategy to improve the likelihood of winning). Once a feasible course of action has been identified, the next step is to plan specific strategies to begin the process of change. Then, to aid with adherence to change during implementation, self-control behaviors are employed. Finally, the cycle is completed with a self-evaluation of the attempted behavior change, the information which is produced then influences the information input of the next cycle (Brown, 1998).

In highly competitive environments such as sport, research suggests that an athlete’s capacity for self-regulation positively influences both their development (McCardle et al., 2018; Young et al., 2023) and performance (Jonker et al., 2010b). McCardle et al. (2018) conducted a survey of 482 athletes’ self-regulated learning in practice, revealed that both junior and senior athletes competing at an international level exhibited significantly higher levels of self-regulation compared to their counterparts participating at the national, provincial, and local levels. Furthermore, Wilson et al. (2021) found that more elite athletes demonstrated superior self-regulated learning during practice sessions. Additionally, Zimmerman (2002) demonstrated that self-regulatory skills accounted for 90% of the variance in volleyball serving skills, highlighting the predictive power of self-regulation in sports performance. In a study conducted by Toering et al. (2009), it was reported that soccer players with enhanced self-regulatory skills were more likely to be classified as being at an elite level. It has been indicated that psychological skills such as self-monitoring and reflection, which constitute integral aspects of the performance and reflection phases of the self-regulatory process, are reported up to 11 times more frequently by elite-level athletes (Bartulovic et al., 2017; Te Wierike et al., 2018). However, despite the considerable amount of research conducted on self-regulation and self-regulated learning in academic and traditional sports contexts, the exploration of self-regulation in esports remains limited.

Researchers acknowledge the potential benefits of investigating self-regulation in esports for players and their social networks (Brevers et al., 2020). A study by Trotter et al. (2021) examined the self-regulatory skills of 993 e’athletes who exhibited low (impaired) to moderate levels of self-reported self-regulatory skills. Furthermore, when compared to other athletic populations, e’athletes displayed notably weaker self-regulatory skills. However, within the sample of e’athletes examined, those belonging to the highest performing group (top 10%) demonstrated significantly higher self-regulatory skills than the lowest performing group (bottom 70%). Although limited, this evidence suggests that like other sporting domains, self-regulatory skills may play a crucial role in esports performance. Research has also indicated differences in self-regulated learning between experts and novice e’athletes (Kleinman et al., 2021). Specifically, in the preparatory phase, novices were found to prioritize process goals more frequently than experts. Additionally, Kleinman et al. (2021) discovered that more advanced League of Legends (LoL) players exhibited greater structure in their practice routines compared to novice LoL players. It was hypothesized that this could be attributed to the design of LoL, as the game itself records players’ progress and provides information, potentially reducing the need for extensive monitoring or self-reflection among LoL players. Furthermore, Kleinman et al. (2022) proposed that digital tools may offer valuable support for e’athletes in implementing their self-regulatory skills. Previous research has shown that psychological skills training (PST) interventions improved athletes’ self-regulatory processes and lead to stressful situations being appraised as a challenge rather than a threat (Hogue, 2020). Further research is warranted to ascertain whether self-regulation has the same potential to influence stress and performance in esports as it does in traditional sports.

### Appraisal

The cognitive-motivational-relational theory (CMRT), proposed by Lazarus (2000), stands as the most influential framework in the field of stress and coping. According to this theory, the process of stress and coping begins with the cognitive appraisal of a stressful event. Lazarus (2000) delineated two distinct types of stress appraisal: primary and secondary appraisal. Primary appraisals revolve around the significance of the outcome of a given stressful situation in relation to the relevance, congruence, and content of a goal. During primary appraisal, an assessment is made regarding the likelihood that the current situation poses a threat to one’s wellbeing. Consequently, the situation is appraised as one of four alternatives: harm/loss (damage that has already occurred), threat (potential future damage), challenge (a sense of enthusiasm about the current or imminent struggle), or benefit (gaining advantage from the situation; see Nicholls and Polman, 2007, for a comprehensive review). Secondary appraisal entails the evaluation of available options and resources that can facilitate coping with the stressor (Lazarus, 2000). It is during secondary appraisal that individuals make decisions regarding which coping strategies to employ (Nicholls and Polman, 2007). Problem-focused and emotion-focused coping methods are often utilized when dealing with stressors appraised as challenges, whereas avoidance-focused coping tends to be employed more frequently in response to stressors perceived as threats (Allen et al., 2012). Threat appraisals are more likely to elicit avoidance coping and emotion-focused strategies (Dias et al., 2012), whereas challenge appraisals are more conducive to the adoption of task-oriented coping strategies (Nicholls et al., 2016). Furthermore, stress appraisal has been found to predict performance outcomes, with challenge appraisals yielding superior performance in training tasks compared to threat appraisals (Gildea et al., 2007).

In recent years, there has been attention given to the study of stress and coping mechanisms in esports. Though, stress appraisal in esports athletes has remained relatively understudied. Poulus et al. (2022b) proposed that the perceived intensity of stressors did not significantly impact the appraisal of stress among League of Legends players. In a separate investigation, Poulus et al. (2020) found that e’athletes were equally likely to perceive stressors as both threats and challenges. Additionally, it was observed that e’athletes tended to view performance-related stressors as challenges, while perceiving challenges arising from teammates as threats (Poulus et al., 2022b). Behnke et al. (2020) discovered that e’athletes who believed they possessed ample personal resources were more likely to achieve favorable outcomes when confronted with stressful situations, compared to those who perceived a lack of personal resources. Leis et al. (2023) suggested that the availability of coping resources and strategies, such as social support, self-regulatory skills, and psychological techniques, could influence the appraisal of competitive situations. Notably, self-regulatory skills, including goal setting, have been found to be associated with stress appraisal, as increased utilization of goal setting has been linked to more effective coping mechanisms and improved mental wellbeing among athletes (Nicholls et al., 2016). Nevertheless, there remains a noticeable dearth of scholarly investigations that have specifically focused on interventions targeting the mitigation of psychological pressure in real-world competitive esport scenarios (Cottrell et al., 2019; Leis et al., 2023). Consequently, there is a pressing need for research that can contribute to the development of evidence-based strategies aimed at effectively managing the challenges posed by performance-related pressure. With this objective in mind, the current study aimed to bridge this gap in knowledge and enhance our comprehension of the influence of appraisal and self-regulation on performance in esports.

## Aims

The primary aim of this study was to investigate potential variations in self-regulation levels among athletes of different levels (national vs. student) and subsequently compare the self-regulation profiles of CS:GO players in the current study to an international sample of e’athletes, utilizing data acquired from previous research (Trotter et al., 2021). Furthermore, the study aimed to assess the predictive capacity of self-regulation on performance outcomes in CS:GO players. The study hypothesized that national-level competitors would exhibit superior self-regulation scores compared to the student group, no significant distinctions would emerge between CS:GO players and the international sample, and self-regulation would serve as a predictor of performance across all e’athletes. Additionally, in an exploratory analysis, considering the absence of prior investigations on the mediating role of self-regulation in the relationship between stress appraisal and performance in esports, the study sought to explore the potential mediational effect of self-regulation in this context among CS:GO players.

## Materials and methods

### Participant information

A total of 53 participants were used as part of the study (*M* age = 21.89, SD = 3.52) consisting of 13.2% females (*n* = 7) and 86.8% males (*n* = 46). Participants were separated based on two criteria: student competitors (SC) who were actively competing in local or University-based competitions and aiming to become national competitors (*n* = 27), consisting of 3 females and 24 males, and national competitors (NC) who were actively aiming to become professional international competitors (*n* = 26) consisting of 4 females and 22 males. The SC participants had in-game ranks which ranged from Distinguished Master Guardian to Global Elite. These participants represented the top 13.99% of all CS:GO players. All NC participants were actively preparing with their teams for ESL Premiership Spring 2022. The ESL premiership is the highest national division of CS:GO in the United Kingdom and Republic of Ireland. The participants, termed national competitors, comprised of four full teams (*n* = 20), including the teams back-up players (*n* = 6). These individuals were salaried only during the competitive season during the time of data collection. The NC participants had in-game ranks which ranged from Supreme Master First Class to Global Elite. These participants represented the top 3.39% of players (96%). However, all but one of the NC participants were in the top 0.75% of all CS:GO players. All individuals held normal or corrected vision and had no known psychiatric or neurological disorders. The study protocol received ethical approval from the United Kingdom-based institution.

### Measures

#### Challenge and threat appraisals (DRES)

The assessment of challenge and threat appraisals involved two items adapted from the cognitive appraisal ratio (Tomaka et al., 1993). The first item measured evaluated task demands through the question “What is your expectation of the demands of the upcoming competition?,” while the second item assessed evaluated personal coping resources by asking “What is your perceived ability to cope with the demands of the upcoming competition?” Both items were rated on a 6-point Likert scale, with response anchors ranging from “Not at all” (scored as 1) to “Extremely” (scored as 6). Following previous research protocols (e.g., Brimmell et al., 2019), a demand-resource evaluation score (DRES) was derived by subtracting the evaluated demands from the evaluated resources. A score of zero or above indicated a challenge appraisal, signifying that personal coping resources matched or exceeded task demands. Conversely, a negative score indicated a threat appraisal, indicating that task demands exceeded personal coping resources.

#### Action performance task

Action performance was assessed by measuring the total time required (in seconds) to complete the CS:GO time-trial and the shooting accuracy percentage in hitting targets. In the context of esports, action performance pertains to attributes such as accuracy that can influence the outcome, such as winning or losing a match (Sharpe et al., 2023a). Each participant completed a single task involving navigating from the starting point to the endpoint of a map while engaging with appearing obstacles. The experimental task was conducted uniformly across all participants, with participants completing four trials with a one-minute break between trails. Consequently, performance variables were averaged, and all individuals followed the same scripted task.

#### Self-regulation questionnaire

The measurement of self-regulatory skills was conducted using the self-regulation questionnaire (SRQ; Brown et al., 1999). Comprising 63 items, the SRQ assesses a global factor and six associated sub-factors. A 5-point Likert scale was employed for scoring, ranging from 1 (strongly disagree) to 5 (strongly agree). While individual factor scores can be computed, it is recommended to utilize an overall SRQ score encompassing all factors (Brown et al., 1999). For classification purposes, overall self-regulation scores can be categorized as high (> 239), intermediate (214–238), or low/impaired (< 213; Brown et al., 1999). The SRQ demonstrates favorable reliability (α = 0.91) and validity (*r* = 0.94, *p* < 0.01) and has been previously employed to measure self-regulation within diverse sporting populations (Brown et al., 1999; Sadri and Janani, 2015; Shandi et al., 2016; Thapar and Nancy, 2018; Gupta and Sudhesh, 2019). The SRQ was found to be highly reliable in the current study (α = 0.95).

### Study design and procedure

Prior to participating in the laboratory experiment, the participants were provided with an information sheet that outlined the study’s objectives and informed them about their ethical entitlements, such as confidentiality, anonymity, and the right to withdraw from the study. Upon granting informed consent, the data collection process commenced and lasted approximately 30 min. The data collection sessions were scheduled during daytime hours (9 am–4 pm), data was gathered by the last-named author (Male), and the participants were instructed to abstain from consuming any beverages containing caffeine for a 24-h period prior to the testing (see Sainz et al., 2020, for discussion). Once the testing process began, participants were initially asked to complete the self-regulation questionnaire, before undergoing a familiarization phase of the domain-specific esport task. Participants were then informed of the primary task, that they were to simply perform to their best ability, and then complete a challenge and threat appraisal assessment (DRES). Following this, they completed four rounds of the performance task, with each round concluding after 3 min if not completed by the participant. A one-minute break was provided between each round. Further detail of the study design and procedure for this experimental study, beyond the inclusion of self-regulation, can be found in Sharpe et al. (2023a). Note: Only the DRES and action performance measures were used in this study, and data was only extracted from the control condition.

### Transparency and openness

The present study involved the extraction of performance data from a subset of the work conducted by Sharpe et al. (2023b), which was then amalgamated with the concurrently collected self-regulation data. Interested parties may request access to the study materials, performance measures, and anonymised data from the corresponding author. Throughout the entire research process, strict adherence was maintained to the American Psychological Association (APA) standards for reporting quantitative research (Appelbaum et al., 2018). The authors hold no conflicts of interest associated with the publication of the following manuscript.

### Statistical analysis

Statistical analyses were conducted using R Studio (RStudio Inc., v 0.99.903) with the R statistical package (v 4.2.1). Assumptions were assessed using the ggplot2 function of the ggpubr package (0.4.0), including evaluations of normality (Fallowfield et al., 2005) and the identification of univariate and multivariate outliers (Kline, 1998). Skewness and Kurtosis values for all measures met the criteria for normality and no outliers were detected. Pearson correlation analysis and a series of paired samples *t*-tests were performed using the rstatix package (0.7.0) to investigate the relationships between challenge and threat appraisals, self-regulation, and performance (see Table 1), and examine potential differences across expertise groups (two levels: student vs. national-level competitors). Participants matchmaking groups then were determined based on the method described prior authors (see Poulus et al., 2020, for information). A one sample *t*-test was then run to compare the self-regulation mean scores from prior literature (Trotter et al., 2020) with SRQ data obtained with permission from the original authors. Finally, mediation analyses were conducted using the PROCESS add-on for SPSS (version 4.30; Hayes and Scharkow, 2013) to explore whether self-regulation mediates the relationship between DRES and action performance (i.e., time trial performance and shooting accuracy). We used 5,000 bias-corrected and accelerated (BCa) bootstrap samples for indirect effects (Davidson and MacKinnon, 2000; Streukens and Leroi-Werelds, 2016). The alpha level (*p*) was set at 0.05 and Cohen’s *d* (with 0.2 being small, 0.5 medium, and 0.8 and above large) were used as effect size estimates (Cohen, 1988).

**TABLE 1:** Demonstrating the differences in variables across levels of expertise.

	Student-level	National-level		
**Variable**	***M* (SD)**	***M* (SD)**	***t* (51)**	**Cohen’s *d***
DRES	−0.65 (2.20)	3.70 (1.14)	9.08***	0.495
Information input	2.47 (0.45)	3.40 (0.67)	5.91***	0.624
Self-evaluation	2.36 (0.62)	3.30 (0.67)	5.21***	0.431
Instigation to change	2.40 (0.51)	3.35 (0.73)	5.39***	0.480
Search	2.41 (0.48)	3.55 (0.48)	8.58***	0.358
Planning	2.46 (0.52)	3.4 (0.59)	6.26***	0.720
Implement	2.27 (0.49)	3.63 (0.53)	9.66***	0.654
Plan evaluation	2.36 (0.55)	3.43 (0.68)	6.24***	0.714
Self-regulation total	150.46 (23.78)	216.85 (30.08)	8.89***	0.443
Time trial performance (s)	77.23 (15.51)	44.14 (3.58)	10.79***	0.966
Accuracy (%)	59 (7.46)	85 (2.56)	17.60***	0.835

*<0.05, **<0.01, and ***<0.001.

## Results

To determine the differences in DRES, self-regulation, and performance scores between levels of expertise a series of *t*-tests were performed (see Table 1).

This study compared the findings obtained in this study with the sample of 993 e’athletes who competed in a variety of esports (Overwatch, League of Legends, Counter-Strike: Global Offensive, Rocket League, and Defense of the Ancients 2), novice to elite (Trotter et al., 2021; see Tables 2–7). When comparing the mean values of self-regulation for the combined study sample, it was found that the study sample was significantly lower on total self-regulation and on all self-regulation subscales. When compared with all skill groups of e’athletes from the Trotter et al. (2021) study, the SC participants scored significantly lower on self-regulation including all subscales of the SRQ. Initially, only the top 10 and 20% skill groups were compared, but each skill group (i.e., the 0–69%, 70–79%, 80–89% and top 10% skill groups) was subsequently examined when significantly lower self-regulation scores were observed between the SC group and all skill groups in the Trotter et al. (2021) study. When comparing the NC group with the top 10% skill group, no differences were observed in total self-regulation, but the NC group was significantly lower on information input, search and implementation.

**TABLE 2:** *T*-test self-regulation comparisons of all participants with the mean of the Trotter et al. (2021) sample.

	*N*	Mean	Comparison M	*t*	sig	Mean diff	Cohen’s *d*	CI
Information input	53	2.95	3.66	−7.05	<0.001***	−0.71	0.74	−0.92/−0.51
Self-evaluation	53	2.84	3.27	−3.93	<0.001***	−0.71	0.80	−0.65/−0.21
Instigation to change	53	2.88	3.48	−5.50	<0.001***	−0.60	0.79	−0.81/−0.38
Search	53	2.99	3.76	−7.46	<0.001***	−0.77	0.75	−0.98/−0.56
Planning	53	2.95	3.20	−2.48	<0.01*	−0.25	0.73	−0.45/−0.05
Implement	53	2.97	3.26	−2.49	<0.01*	−0.29	0.85	−0.53/−0.06
Plan evaluation	53	2.91	3.42	−4.55	<0.001***	−0.51	0.82	−0.74/−0.29
Total SR	53	184.28	216.45	−5.45	<0.001***	−32.17	42.98	−44.01/−20.32

* < 0.05, ** < 0.01, *** < 0.001.

**TABLE 3:** *T*-test self-regulation comparisons of the SC group with the 0–69% group of the Trotter et al. (2021) sample.

	*N*	Mean	Comparison M	*t*	sig	Mean diff	Cohen’s *d*	CI
Information input	26	2.47	3.63	−12.98	<0.001***	−1.16	0.45	−1.34/−0.97
Self-evaluation	26	2.36	3.27	−7.35	<0.001***	−0.91	0.63	−1.16/−0.65
Instigation to change	26	2.41	3.44	−10.27	<0.001***	−1.03	0.51	−1.24/−0.83
Search	26	2.41	3.72	−13.73	<0.001***	−1.31	0.49	−1.51/−1.12
Planning	26	2.46	3.15	−6.68	<0.001***	−0.69	0.53	−0.90/−0.48
Implement	26	2.28	3.26	−10.09	<0.001***	−0.98	0.50	−1.18/−0.78
Plan evaluation	26	2.36	3.39	−9.47	<0.001***	−1.03	0.55	−1.25/−0.80
Total SR	26	150.46	214.68	−13.77	<0.001***	−64.22	23.78	−73.83/−54.61

*** < 0.001.

**TABLE 4:** *T*-test self-regulation comparisons of the SC group with the 70–79% group of the Trotter et al. (2021) sample.

	*N*	Mean	Comparison M	*t*	sig	Mean diff	Cohen’s *d*	CI
Information input	26	2.47	3.65	−13.21	<0.001***	−1.17	0.45	−1.36/−0.99
Self-evaluation	26	2.36	3.36	−8.08	<0.001***	−1.00	0.63	−1.25/−0.74
Instigation to change	26	2.41	3.52	−11.06	<0.001***	−1.11	0.51	−1.32/−0.91
Search	26	2.41	3.77	−14.26	<0.001***	−1.36	0.49	−1.56/−1.17
Planning	26	2.46	3.21	−7.26	<0.001***	−0.75	0.53	−0.96/−0.54
Implement	26	2.28	3.24	−9.89	<0.001***	−0.96	0.50	−1.16/−0.76
Plan evaluation	26	2.36	3.49	−10.39	<0.001***	−1.23	0.55	−1.35/−0.90
Total SR	26	150.46	218.21	−14.52	<0.001***	−67.75	23.78	−77.35/−58.14

*** < 0.001.

**TABLE 5:** *T*-test self-regulation comparisons of the SC group with the 80–89% group of the Trotter et al. (2021) sample.

	*N*	Mean	Comparison M	*t*	sig	Mean diff	Cohen’s *d*	CI
Information input	26	2.47	3.75	−14.33	<0.001***	−1.28	0.45	−1.46/−1.09
Self-evaluation	26	2.36	3.24	−7.11	<0.001***	−0.88	0.63	−1.13/−0.62
Instigation to change	26	2.41	3.56	−11.46	<0.001***	−1.15	0.51	−1.36/−0.95
Search	26	2.41	3.74	−13.94	<0.001***	−1.33	0.49	−1.53/−1.14
Planning	26	2.46	3.25	−7.65	<0.001***	−0.79	0.53	−1.00/−0.58
Implement	26	2.28	3.29	−10.40	<0.001***	−1.01	0.50	−1.21/−0.81
Plan evaluation	26	2.36	3.41	−9.65	<0.001***	−1.05	0.55	−1.27/−0.82
Total SR	26	150.46	218.29	−14.54	<0.001***	−67.82	23.78	−77.44/−58.22

*** < 0.001.

**TABLE 6:** *T*-test self-regulation comparisons of the SC group with the top 10% group of the Trotter et al. (2021) sample.

	*N*	Mean	Comparison M	*t*	sig	Mean diff	Cohen’s *d*	CI
Information input	26	2.47	3.81	−15.00	<0.001***	−1.34	0.45	−1.52/−1.15
Self-evaluation	26	2.36	3.33	−7.84	<0.001***	−0.97	0.63	−1.22/−0.71
Instigation to change	26	2.41	3.50	−10.86	<0.001***	−1.09	0.51	−1.30/−0.89
Search	26	2.41	3.93	−15.93	<0.001***	−1.52	0.49	−1.72/−1.33
Planning	26	2.46	3.37	−8.81	<0.001***	−0.91	0.53	−1.12/−0.70
Implement	26	2.28	3.40	−11.53	<0.001***	−1.12	0.50	−1.32/−0.92
Plan evaluation	26	2.36	3.51	−10.58	<0.001***	−1.15	0.55	−1.37/−0.92
Total SR	26	150.46	223.59	−15.68	<0.001***	−73.13	23.78	−82.74/−63.52

*** < 0.001.

**TABLE 7:** *T*-test self-regulation comparisons of the NC group with the top 10% group of the Trotter et al. (2021) sample.

	*N*	Mean	Comparison M	*t*	sig	Mean diff	Cohen’s *d*	CI
Information input	27	3.40	3.81	−3.17	<0.01**	−0.41	0.67	−0.67/−0.14
Self-evaluation	27	3.29	3.33	−0.26	= 0.40	−0.03	0.67	−0.30/0.23
Instigation to change	27	3.34	3.50	−1.09	= 0.14	−0.15	0.73	−0.44/0.14
Search	27	3.55	3.93	−4.06	<0.001***	−0.38	0.49	−0.57/−0.18
Planning	27	3.42	3.37	0.44	= 0.33	0.05	0.59	−0.18/−0.28
Implement	27	3.63	3.40	2.32	= 0.01*	0.23	0.52	0.03/0.44
Plan evaluation	27	3.43	3.51	−0.59	= 0.28	−0.08	0.68	−0.35/0.19
Total SR	27	216.85	223.59	−1.16	= 0.13	−6.73	30.08	−18.64/5.16

* < 0.05, ** < 0.01, *** < 0.001.

**FIGURE 1:**
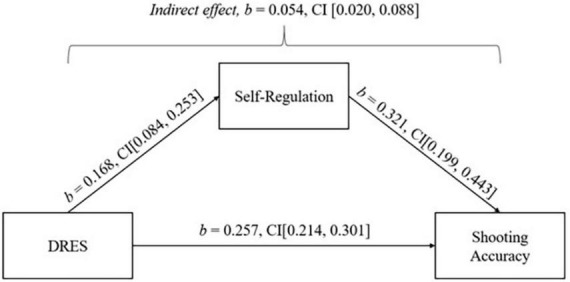
Simple mediation model: Self-regulation mediating the relationship between mental demand and accuracy.

Two simple mediation analyses, following Baron and Kenny (1986) three step method, were performed to investigate whether self-regulation total significantly mediates the relationship between DRES and performance. First, self-regulation significantly mediated the relationship between DRES and shooting accuracy. The total effect of the model was significant [*b* = 0.312, 95% CI (0.265, 0.369)]. Direct effects [*b* = 0.257, 95% CI (0.214, 0.301)] and indirect effects were found to be statistically significant [*b* = 0.054, 95% CI (0.020, 0.088)]. See Figure 1 for path coefficients.

**FIGURE 2:**
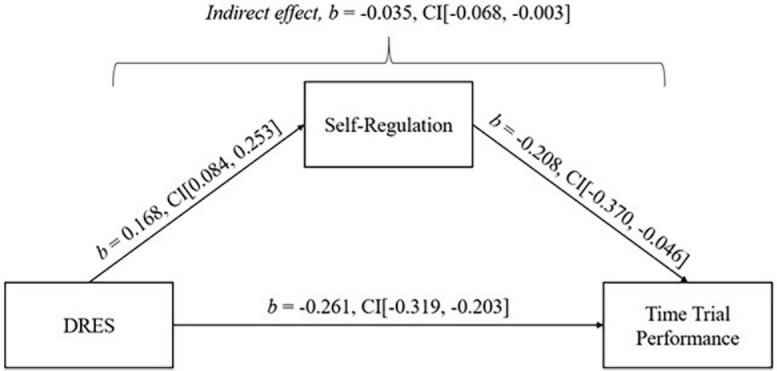
Simple mediation model: Self-regulation mediating the relationship between mental demand and time performance.

Second, self-regulation significantly mediated the relationship between DRES and time trial performance. The total effect of the model was significant [*b* = −0.296, 95% CI (−0.350, −0.242)]. Direct effects [*b* = −0.261, 95% CI (−0.319, −0.203)] and indirect effects were found to be statistically significant [*b* = −0.035, 95% CI (−0.068, −0.003)]. See Figure 2 for path coefficients.

## Discussion

This study aimed to explore the variations in self-regulation among NC and SC CS:GO players, compared their self-regulation results with an international sample, and investigated self-regulation’s predictive role in performance. It also explored self-regulation as a potential mediator between stress appraisal and performance. The findings indicated that the examined group of e’athletes demonstrated compromised or borderline compromised levels of self-regulation. However, the NC group exhibited significantly higher self-regulation than the SC group. When comparing the self-regulation of CS:GO players to a global sample of e’athletes, it was observed that the CS:GO players exhibited significantly weaker self-regulatory skills than the international population (Trotter et al., 2021). Furthermore, the NC group outperformed the SC group in terms of shooting accuracy and time trial performance. In addition, the NC group displayed a greater tendency to perceive stressful situations as a challenge rather than a threat compared to the SC group. Finally, self-regulation was found to mediate the relationship between stress appraisal and both shooting accuracy and time trial performance. Based on these findings, we contend that despite the impaired or borderline impaired self-regulation reported by the e’athlete sample in this study, individuals with superior self-regulation abilities were more likely to achieve better performance outcomes. We propose that this may be attributed to the mediating effect of self-regulation between stress appraisal and the action performance metrics.

### Self-regulation

Initial data suggested that the NC group exhibited self-regulation skills at the lower end of the moderate range, with only borderline scores (i.e., a mean score only two points above the impaired classification cutoff) between impaired and moderate self-regulation being reported. Nonetheless, this finding corroborated earlier research, which found that e’athletes reported borderline moderate/impaired-level self-regulation skills (Trotter et al., 2021). On the other hand, the SC group demonstrated impaired levels of self-regulation skills, indicating a lower proficiency compared to the global sample that identified impaired to moderate levels of self-regulation among all skill groups. Similar patterns have been observed in other sports, where self-regulatory abilities have been identified as a differentiating factor between elite football players and their less skilled counterparts (Toering et al., 2009). Additionally, a study focusing on women’s volleyball serving skills revealed that experts exhibited superior self-regulation abilities compared to non-experts and novices. Furthermore, self-regulation was found to account for 90% of the variance in serving skills (Kitsantas and Zimmerman, 2002). Consequently, it is recommended that athletes, coaches, and professional esports organizations aiming to enhance performance prioritize the development of psychological skills that contribute to improved self-regulation (McCrory et al., 2013). This may be especially important for CS:GO players, as the SC group whose online ranks represent the top 14% of CS:GO players reported significantly lower self-regulation than the lowest skill groups from the international sample collected by Trotter et al. (2021). However, the authors wish to note that prior literature has also observed inconsistencies associated with in-game rank influencing variables of interest (Toth et al., 2019). Such findings may be unsurprising given that in-game rank is merely an indication of success with temporary teams, like that of other popular competitive esports (Kou et al., 2016), and not necessarily a direct reflection of an individual’s performance capability. For example, in CS:GO’s in-game matchmaking system, teams are collated at random to compete in a best-of-30 rounds for a period of 45–60 min. Teams may, or may not, communicate through verbal, typing, or in-game signals to surpass the performance of their opponents. Given CS:GO requires some degree of team cohesion, and that negative team communication has been associated with poor performance (Kwak et al., 2015), it is unlikely a single individual can “carry” (referring to an individual’s capacity to independently achieve success) their entire team to victory. As such, caution should be taken when using in-game rank as a direct classification method of an individual’s performance capacity, and so should be avoided when possible.

When examining self-regulation in comparison to a global population sample, the SC group demonstrated significantly lower levels of self-regulation across all subscales, in contrast to all comparison skill groups. The NC group exhibited significantly lower scores in informational input, search, and implementation when compared to the top 10% skill group in the global sample (Trotter et al., 2021). Notably, both the NC and SC groups exhibited notably inferior information gathering skills, which form part of the information input phase of self-regulation, in comparison to the global sample of e’athletes. This may affect stress appraisal as the information input phase initiates the self-regulation cycle, and may occur while the individual appraises the stressful situation and subsequently influences which coping strategy an individual employs. Considering that self-regulation has been found to be associated with success across various performance domains, such as sports and academia (Jonker et al., 2010a), it is disconcerting that both the SC and NC groups scored significantly lower than the global sample of e’athletes.

### Stress appraisal

Regarding the appraisal process, it was observed that the NC group demonstrated a greater tendency to appraise situations as challenges, while the SC group tended to perceive situations as threats. This discrepancy may be attributed to several factors. Firstly, the NC group reported significantly higher levels of in-game skills, such as accuracy and time trial performance, as well as self-regulation than the SC group. Additionally, the NC group had access to additional resources, such as coaches and organizational support. These internal and external personal and perceived resources may have resulted in the CS:GO players appraisal of stressful situations as challenges. According to the Lazarus (2000) when a person has access to both internal (e.g., psychological or self-regulatory skills; Nicholls et al., 2016) and external resources (e.g., social support; Pakenham et al., 2007) are available they are more likely to appraise stress as a challenge rather than a threat. Nevertheless, study results indicate that skill level and the availability of internal and external resources may be linked to stress appraisal among e’athletes. While there is limited existing literature that supports such claims, this in fact falls in accordance with the Biopsychosocial Model of Challenge and Threat (Blascovich, 2008), which tentatively proposes that factors such as skill/expertise influence appraisal evaluations, thus facilitating the maintenance of performance. Coaches, organizations, and support systems (e.g., grassroots esports clubs) within the esports industry should, in addition to developing player experience (e.g., tournament experience, in-game strategy), consider implementing strategies to help e’athletes develop positive stress appraisal patterns, as this will likely impact the coping strategies e’athletes use to deal with stressful situations which impact their performance and wellbeing.

### Mediation

Mediation analysis revealed that self-regulation played a significant mediating role in the relationship between stress appraisal and performance outcomes (i.e., accuracy and time trial performance). The results indicated a direct effect of stress appraisal on both performance factors, with appraising a stressful situation as a challenge being associated with higher accuracy and faster time trial performance. Moreover, the indirect effects demonstrated that self-regulation mediated the relationship between stress appraisal and performance outcomes, whereby higher self-regulation explained the performance outcomes when e’athletes appraised the stressful situation as a challenge. To the authors’ knowledge, this study represents the first examination of the mediating effect of self-regulation between stress appraisal and performance factors. One possible explanation for this mediating effect is that e’athletes who perceive a stressful situation as a challenge possess better self-regulatory skills, allowing them to adopt problem-focused coping strategies to directly deal with the problems which are causing their stress. The current study tentatively suggests that enhancing self-regulatory skills may contribute to better performance in terms of accuracy and time trial results in esports. This would imply that interventions targeting self-regulation could positively impact the overall performance of e’athletes.

### Practical applications and limitations

The incorporation of psychological skills, such as goal setting and imagery, into the training regimen of e’athletes is worthy of consideration for the purpose of fostering self-regulation. Particularly for novice e’athletes, who demonstrated impaired self-regulatory abilities, these psychological skills hold potential for substantial benefits. While further investigation specific to e’athletes is warranted, existing research has recognized an associated link between self-regulatory skills and academic as well as sports performance (Kitsantas and Zimmerman, 2002; Jonker et al., 2010a; Trotter et al., 2021). Ideally, the cultivation of these skills should commence at the grassroots level, wherein disparities between student e’athletes and their peers are minimal (Trotter et al., 2022). By integrating these skills early in their development, novice e’athletes can establish a solid foundation for self-regulation, facilitating their progress as they advance in their competitive journey (Trotter et al., 2022). Furthermore, incorporating these psychological techniques into coaching and training programs can help establish a holistic framework that nurtures the mental and emotional wellbeing of e’athletes. Particularly, when literature has demonstrated that the arduous nature of competitive gaming can indeed exact a toll on the mental and emotional wellbeing of e’athletes, resulting in heightened pressure, mental ill health symptoms, and burnout (Smith et al., 2022). It is therefore imperative to provide e’athletes with a repertoire of self-regulatory strategies, empowering them to skilfully navigate stress, sustain concentration, and enhance decision-making amidst high-pressure situations. By honing these self-regulatory skills, e’athletes can not only optimize their performance but also cultivate resilience, bolster psychological wellbeing, and experience greater overall satisfaction within their chosen pursuit (Leis et al., 2023).

This study presents certain limitations that warrant acknowledgment. Foremostly the sample size utilized in this research is relatively small, comprising solely one esports discipline within a specific geographical region. Consequently, the generalizability of these findings to other esports or larger cohorts of e’athletes remains uncertain. Cultural, socioeconomic, and environmental factors inherent to the geographical region (UK and the Republic of Ireland) where participants were recruited could have influenced the results in ways that may not be applicable to e’athletes from diverse backgrounds or alternative gaming ecosystems. Therefore, caution should be exercised when attempting to extrapolate these findings to a broader population of e’athletes or when comparing them across different regions or cultural contexts.

Additionally, the study’s focus on one esports discipline limits its applicability to variations in self-regulatory processes across different games. Previous research has suggested that different genres of video games may promote development of different cognitive skills (Granic et al., 2014). However, differences in self-regulatory skills between genres of esports has not yet been explored and may offer an interesting future area of research. Future research should address these limitations by including larger and more diverse samples encompassing multiple esports disciplines and participants from various regions. This may enhance understanding of self-regulatory processes in esports and aid in developing tailored interventions and training programs to support the psychological wellbeing and performance optimization of e’athletes across different contexts.

Furthermore, it has been posited that primary and secondary appraisal constitute integral components of the preparatory phase of self-regulation (Panadero, 2017). However, this proposition is confined to the dual processing model (Boekaerts and Cascallar, 2006), while more widely employed models of self-regulation (Zimmerman, 2000) do not explicitly incorporate the concepts of primary and secondary appraisal. If stress appraisal indeed forms an integral part of the self-regulation process, it may provide an explanation for the substantial correlation observed between appraisal and self-regulation. Therefore, it is recommended that future research delves deeper into the intricate relationship between stress appraisal and the preparatory phases of self-regulation.

Another limitation is that the present study did not differentiate between different types of self-regulation (e.g., behavioral, emotional). As a result, we have not explored the effectiveness of other interventions which could be used to target the development of specific forms of self-regulation. For example, although PST is known to foster behavioral self-regulation (Ntoumanis and Cumming, 2016), other interventions such as acceptance and commitment therapy (ACT) have been shown to improve emotional regulation (Mahmoudpour et al., 2021). Future studies should explore how different psychological interventions might influence the development of specific types of self-regulation in esports.

Finally, current research exploring regulation in esports has focused on the individual athlete. However, as many esports games occur in teams future research should explore how cognitive and behavioral regulation occurs in teams in esports. Previous research has reported that teammates represent a large proportion of stressors for e’athletes (Poulus et al., 2022a). How teams collectively regulate their behaviors may give further insight new methods for minimizing the stress cause by teammate interactions.

## Conclusion

This study reveals that CS:GO players exhibited impaired self-regulation compared to a global sample of e’athletes. However, CS:GO players with better self-regulatory skills outperformed their peers in shooting accuracy and time trial performance. Better self-regulation was also associated with the appraisal of stressful situations as challenges, while lower self-regulation was associated with threat appraisal. These findings align with previous research in sports, emphasizing the importance of psychological skills development to enhance self-regulation and improve esports performance. It is recommended that athletes, coaches, and organizations in esports prioritize the cultivation of psychological skills that contribute to improved self-regulation. It may be beneficial to have to have a sport psychologist support the introduction or augmentation of psychological skills into the performance environment, especially if e’athletes are at risk of, or suffer from mental ill health. Additionally, this study highlights the need for interventions targeting information input skills, essential for effective coping and initiating self-regulation. Furthermore, self-regulation was found to mediate the relationship between stress appraisal and performance, indicating that better self-regulation leads to improved shooting accuracy and faster time trial performance. This suggests that e’athletes with higher self-regulatory skills perceive themselves as having more personal resources, enabling them to appraise challenging situations and utilize problem-focused coping strategies. Overall, this research underscores the significance of self-regulation in esports performance and provides valuable insights for player development, action performance, and outcomes in the field.

## Data availability statement

The raw data supporting the conclusions of this article will be made available by the authors, without undue reservation.

## Ethics statement

The studies involving humans were approved by the University of Chichester Research Ethics Committee. The studies were conducted in accordance with the local legislation and institutional requirements. The participants provided their written informed consent to participate in this study.

## Author contributions

MT: Conceptualization, Data curation, Methodology, Project administration, Writing—original draft, Writing—review and editing. EO: Data curation, Formal analysis, Methodology, Writing—review and editing. BS: Conceptualization, Data curation, Formal analysis, Investigation, Methodology, Project administration, Writing—original draft, Writing—review and editing.
